# Renal Cortical Glucose Uptake Is Decreased in Insulin Resistance and Correlates Inversely With Serum Free-fatty Acids

**DOI:** 10.1210/clinem/dgad663

**Published:** 2023-11-13

**Authors:** Eleni Rebelos, Andrea Mari, Miikka-Juhani Honka, Laura Pekkarinen, Aino Latva-Rasku, Sanna Laurila, Johan Rajander, Paulina Salminen, Hidehiro Iida, Ele Ferrannini, Pirjo Nuutila

**Affiliations:** Turku PET Centre, University of Turku, 20520, Turku, Finland; Department of Clinical and Experimental Medicine, University of Pisa, Pisa, 56126, Italy; InFLAMES Research Flagship, University of Turku, 20014, Turku, Finland; CNR Institute of Neuroscience, Padova, 35121, Italy; Turku PET Centre, University of Turku, 20520, Turku, Finland; InFLAMES Research Flagship, University of Turku, 20014, Turku, Finland; Division of Information Science, Nara Institute of Science and Technology, Takayamacho 8916-5, Ikoma, Nara 630-0192, Japan; Turku PET Centre, University of Turku, 20520, Turku, Finland; Department of Endocrinology, Turku University Hospital, 20521, Turku, Finland; Turku PET Centre, University of Turku, 20520, Turku, Finland; Department of Endocrinology, Turku University Hospital, 20521, Turku, Finland; Turku PET Centre, University of Turku, 20520, Turku, Finland; Heart Center, Turku University Hospital, 20521, Turku, Finland; Department of Medicine, University of Turku, 20520, Turku, Finland; Turku PET Centre, Accelerator Laboratory, Åbo Akademi University, 20521, Turku, Finland; Division of Digestive Surgery and Urology, Turku University Hospital, 20521, Turku, Finland; Turku PET Centre, University of Turku, 20520, Turku, Finland; CNR Institute of Clinical Physiology, Pisa, 56124, Italy; Turku PET Centre, University of Turku, 20520, Turku, Finland; InFLAMES Research Flagship, University of Turku, 20014, Turku, Finland; Department of Endocrinology, Turku University Hospital, 20521, Turku, Finland

**Keywords:** positron emission tomography, kidney, metabolism, obesity, insulin resistance

## Abstract

**Context:**

Studies on human renal metabolism are scanty. Nowadays, functional imaging allows the characterization of renal metabolism in a noninvasive manner. We have recently demonstrated that fluorodeoxyglucose F18 (^18^F FDG) positron emission tomography can be used to analyze renal glucose uptake (GU) rates, and that the renal cortex is an insulin-sensitive tissue.

**Objective:**

To confirm that renal GU is decreased in people with obesity and to test whether circulating metabolites are related to renal GU.

**Design, Setting and Participants:**

Eighteen people with obesity and 18 nonobese controls were studied with [^18^F]FDG positron emission tomography during insulin clamp. Renal scans were obtained ∼60 minutes after [^18^F]FDG injection. Renal GU was measured using fractional uptake rate and after correcting for residual intratubular [^18^F]FDG. Circulating metabolites were measured using high-throughput proton nuclear magnetic resonance metabolomics.

**Results:**

Cortical GU was higher in healthy nonobese controls compared with people with obesity (4.7 [3.4-5.6] vs 3.1 [2.2-4.3], *P* = .004, respectively), and it associated positively with the degree of insulin sensitivity (M value) (*r* = 0.42, *P* = .01). Moreover, cortical GU was inversely associated with circulating β-OH-butyrate (*r* = -0.58, *P* = .009), acetoacetate (*r* = -0.48, *P* = .008), citrate (*r* = −0.44, *P* = .01), and free fatty acids (*r* = −0.68, *P* < .0001), even when accounting for the M value. On the contrary, medullary GU was not associated with any clinical parameters.

**Conclusion:**

These data confirm differences in renal cortical GU between people with obesity and healthy nonobese controls. Moreover, the negative correlations between renal cortex GU and free fatty acids, ketone bodies, and citrate are suggestive of substrate competition in the renal cortex.

Studies of renal physiology in humans have traditionally been hampered by the functional heterogeneity of the renal cells and the marked differences in perfusion and oxygenation between the cortex and the medulla. More recently, however, advancements in medical imaging have made significant headway toward thus-far poorly studied aspects of renal physiology in humans in vivo. Recent elegant studies have applied magnetic resonance imaging to the assessment of renal perfusion and oxygenation (1). Reliable measurements of renal perfusion are also obtained with the use of positron emission tomography (PET) (2, 3), which makes it possible to simultaneously determine regional renal substrate uptake rates.

With regard to abnormalities of renal metabolism in obesity, it is well established that obese individuals have higher total renal perfusion compared with lean controls (4), a hemodynamic alteration that contributes to the enhanced glomerular filtration rate that is characteristic of obesity (5-7). Moreover, in a previous PET study using 14(R,S)-F 18 (^18^F)fluoro-6-thia-heptadecanoate to trace renal free fatty acid (FFA) uptake, we have shown that people with obesity have enhanced cortical and medullary FFA uptake rates compared with lean controls (4). The assessment of renal glucose uptake (GU) has mostly relied on catheterization studies of arteriovenous concentration differences, which have yielded highly variable estimates of total renal GU. We have recently described a novel approach whereby regional renal GU can be reliably estimated using [^18^F]fluorodeoxyglucose (FDG)-PET provided that tissue [^18^F]FDG activity is corrected for the residual intratubular [^18^F]FDG activity as measured at late acquisition times (8). By this method, we showed that the “corrected” cortical and medullary GU were both higher in healthy lean controls than in people with obesity under fasting as well as hyperinsulinemic euglycemic clamp conditions. Moreover, cortical, but not medullary GU, was stimulated during the clamp period. The conclusion that the human renal cortex is an insulin-sensitive tissue is in line with previous findings that have shown the presence of insulin receptors in the renal cortex (9), and arteriovenous differences experiments that reported higher renal GU during insulin clamp (10).

Aims of the present study were to assess whether there are differences in renal cortex GU rates in a larger sample of people with obesity and healthy nonobese controls under insulin clamp conditions. Moreover, because the kidney can use several substrates for its metabolic needs (11), we measured circulating metabolites and FFA and assessed whether there are associations between renal glucose uptake and circulating metabolites.

## Methods

### Participants and Study Design

Eighteen people with severe obesity and 18 age- and sex-matched healthy, nonobese controls were studied. People with obesity were candidates for bariatric surgery and were recruited from the Division of Digestive Surgery of Turku University Hospital, Turku, Finland. Healthy nonobese controls were recruited via advertisements in the local newspapers.

For the obese group, inclusion criteria were: age 18 to 60 years, body mass index (BMI) 35 to 45 kg/m^2^ or BMI 32 to 45 kg/m^2^, and diagnosed type 2 diabetes (T2D); exclusion criteria were: presence of metal objects in the body, previous participation in PET studies, pregnancy, poor compliance, alcohol or drug abuse, T2D with fasting glucose levels ≥7.0 mmol/L, or treatment with insulin, and any chronic disease, medication, or condition that could create a hazard to subject safety, endanger study procedures, or interfere with the interpretation of results. For the healthy nonobese group, inclusion criteria were: age 18 to 60 years, BMI 18 to 28 kg/m^2^, and absence of T2D; exclusion criteria were as those for people with obesity, plus history of eating disorders and drastic weight grain or weight loss.

All study participants underwent a screening visit in which written informed consent was provided before participating in the study and information regarding medical history and drug use was collected. Blood pressure was measured 3 times with OMRON 711 automatic blood pressure monitor (Omron Corporate, Kyoto, Japan) in subjects seated for at least 10 minutes, and the average of the last 2 measurements was recorded. Fasting blood and urine samples were collected and a standard (75-g) oral glucose tolerance test with frequent blood sampling every 30 minutes was performed. According to the American Diabetes Association criteria (12), 4 people with obesity had T2D; of them, 1 was on metformin and 3 were on a combination of metformin and SGLT2 inhibitors. All antidiabetic drugs were suspended 3 days before the metabolic studies. The anthropometric and clinical characteristics of the study participants are listed in Table 1. The study protocol was approved by the Ethics Committee of the Hospital District of Southwestern Finland (ClinicalTrials.gov: NCT04343469).

**Table 1.** Clinical and metabolic characteristics of the study subjects

	Nonobese	Obese	*P*
M/W	5/13	5/13	>.9
Age (y)	45 ± 11	49 ± 9	.3
Systolic BP (mm Hg)	118 [113-130]	138 [127-147]	.001
Diastolic BP (mm Hg)	79 [72-85]	84 [77-90]	.05
Body weight (kg)	69 [56-79]	107 [97-133]	<.0001
BMI (kg/m^2^)	23.2 [21.7-25.3]	39.0 [36.5-42.0]	<.0001
NGT/IFG and IGT/T2D	16/2/0	7/7/4	.006
HbA_1c_ (mmol/mol)	34 [31-36]	37 [33-39]	.03
M value (µmol/min^−1^/kg^−1^)	45 [39-53]	16 [7-23]	<.0001
Estimated glomerular filtration rate (mL/min/1.73 m^2^)	94 [83-98]	97 [84-104]	.5
Urinary ACR (mg/mmol)	0.4 [0.4-0.6]	0.4 [0.4-0.7]	.3
Fasting plasma glucose (mmol/L)	5.1 ± 0.4	5.8 ± 0.7	.003
Clamp plasma glucose (mmol/L)	5.2 ± 0.3	5.1 ± 0.2	.7
Clamp plasma insulin (pmol/L)	417 [339-472]	484 [407-545]	.06
Fasting serum FFA (mmol/L)	0.47 [0.37-0.55]	0.59 [0.49-0.65]	.03
Clamp serum FFA (mmol/L)	0.03 [0.02-0.05]	0.11 [0.06-0.16]	<.0001
ß-OH-butyrate (mmol/L)	0.001 [0-0.006]	0.004 [0-0.013]	.3
Acetoacetate (mmol/L)	0.010 [0.006-0.015]	0.011 [0.007-0.018]	.3
Citrate (mmol/L)	0.06 [0.05-0.07]	0.07 [0.06-0.07]	.04

Entries are mean ± SD or median [interquartile range]. Clamp plasma insulin and clamp plasma glucose levels during the kidney scan are shown. *P* value for the difference between obese and nonobese individuals.

Abbreviations: ACR, albumin-to-creatinine ratio; BMI, body mass index; BP, blood pressure; FFA, free fatty acid; HbA_1c_, glycated hemoglobin; IFG, impaired fasting glucose; IGT, impaired glucose tolerance; M, men; NGT, normal glucose tolerance; T2D, type 2 diabetes; W, women.

### Study Protocol

After an overnight fast (10-12 hours), study participants arrived at the facilities of the Turku PET Centre, Turku, Finland. After voiding, patients had 2 catheters inserted in the antecubital veins of each arm, 1 for the administration of radiolabeled tracer, glucose, and insulin, and the other for arterialized blood sampling. The gold standard method for the assessment of insulin resistance (ie, euglycemic hyperinsulinemic clamp) was started as previously described (13). In brief, a primed-continuous insulin infusion was given at a rate of 40 mU/m^−2^/min^−1^, followed by a variable 20% dextrose infusion to raise plasma insulin. Because the aim of this experiment is to “clamp” the plasma glucose levels at ∼4.5 to 5.5 mmol/L, a higher glucose infusion rate is indicative of better insulin sensitivity. Blood samples were drawn every 5, 30, and 60 minutes for the determination of plasma glucose, insulin, and FFA, respectively. Sixty minutes into the clamp, subjects were positioned inside the PET scanner, 181 ± 9 MBq of [^18^F]FDG was injected, and radioactivity acquisition was started. Renal radioactivity was measured at 60 to 70 minutes after injection. In 6 healthy nonobese participants and 1 obese participant, the acquisition of renal radioactivity was anticipated at ∼45 minutes from [^18^F]FDG injection because of technical issues. During the scan, the arm used for venous blood sampling was warmed with a heating pillow throughout the clamp study to obtain arterialized venous blood every 5 minutes for the determination of plasma radioactivity, as in previous studies (14, 15). Immediately after the completion of the PET scanning, subjects voided their bladders for the measurement of [^18^F]FDG excreted in the urine. Plasma and urinary radioactivity concentrations were measured using an automatic γ-counter (Wizard 1480; Wallac, Turku, Finland).

### PET Data Analysis

**Figure 1.**
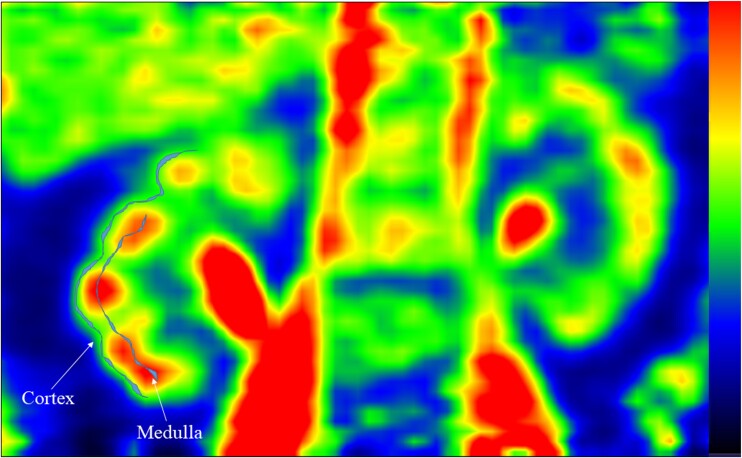
Representative example of ROI placement in the renal cortex and medulla.

PET images were reconstructed in a 256 × 256 matrix after correction for decay time, dead time, and photon attenuation. Image analysis was performed using Carimas v.2.9 (http://www.turkupetcentre.fi/). To obtain the time-radioactivity curves, the regions of interest (ROIs) were manually drawn on PET/computed tomography fusion images in the renal cortex and medulla. In particular, after fixing the activity scale for all subjects, 4 to 5 consecutive thin ROIs were drawn in the region of slightly lower radioactivity just outside the high signal coming from the renal pyramids; this ROI was considered to represent the renal cortex (Fig. 1). A second thin ROI was drawn more centrally on the same slices, representing the medulla (Fig. 1). Regional renal glucose metabolism was assessed using fractional uptake rate (FUR), as described previously (8). In brief, we used the individual urine radioactivity values to calculate late tubular [^18^F]FDG radioactivity within the ROI to correct regional [^18^F]FDG uptake rates by subtracting late tubular [^18^F]FDG radioactivity from the ROI radioactivity (8). To obtain GU rates, FUR (1/min) was multiplied by plasma glucose concentration. GU rates are expressed in µmol/min per 100 mL of tissue (µmol/min^−1^/100 mL^−1^). Because the lumped constant for the kidneys is not known, it was assumed to be 1.

### Calculations

Insulin-stimulated glucose disposal (M value) was used as a measure of whole-body insulin sensitivity, as previously described (16). The M value was calculated using the glucose infusion rates during the second hour of the clamp as a mean of three 20-minute intervals. Mean arterial pressure was calculated as the diastolic blood pressure plus one third of the pulse pressure. Estimated glomerular filtration rate was calculated by the Chronic Kidney Disease Epidemiology Collaboration equation.

### Biochemical Analyses

Plasma glucose was determined in duplicate by the glucose oxidase method (Analox GM9, Analox Instruments, London, UK). Serum insulin concentration was measured by an automatized electro-chemiluminescence immunoassay (Cobas e601, Roche Diagnostics GmbH, Mannheim, Germany). Serum FFA concentration was determined using an enzymatic assay (ACS-ACOD, Wako Chemicals GmbH, Neuss, Germany) on a Cobas c702 automatic analyzer (Roche Diagnostics GmbH).

### Serum NMR Metabolomics

Serum metabolic biomarkers were quantified during the insulin clamp (∼120 minutes from the start) using high-throughput proton nuclear magnetic resonance (NMR) metabolomics (Nightingale Health Ltd, Helsinki, Finland). This method provides simultaneous quantification of routine lipids, fatty acid composition, and various low-molecular metabolites including amino acids, ketone bodies, and gluconeogenesis-related metabolites in molar concentration units and lipoprotein subclass profiling with lipid concentrations within 14 subclasses. The NMR metabolomics platform has been described previously (17).

### Statistical Analysis

**Figure 2.**
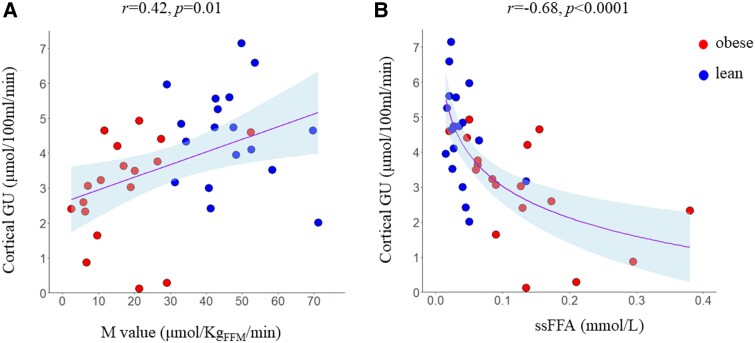
Cortical glucose uptake was positively associated with the M value (A), and inversely with the FFA levels measured during the steady-state period of the insulin clamp (B).

**Figure 3.**
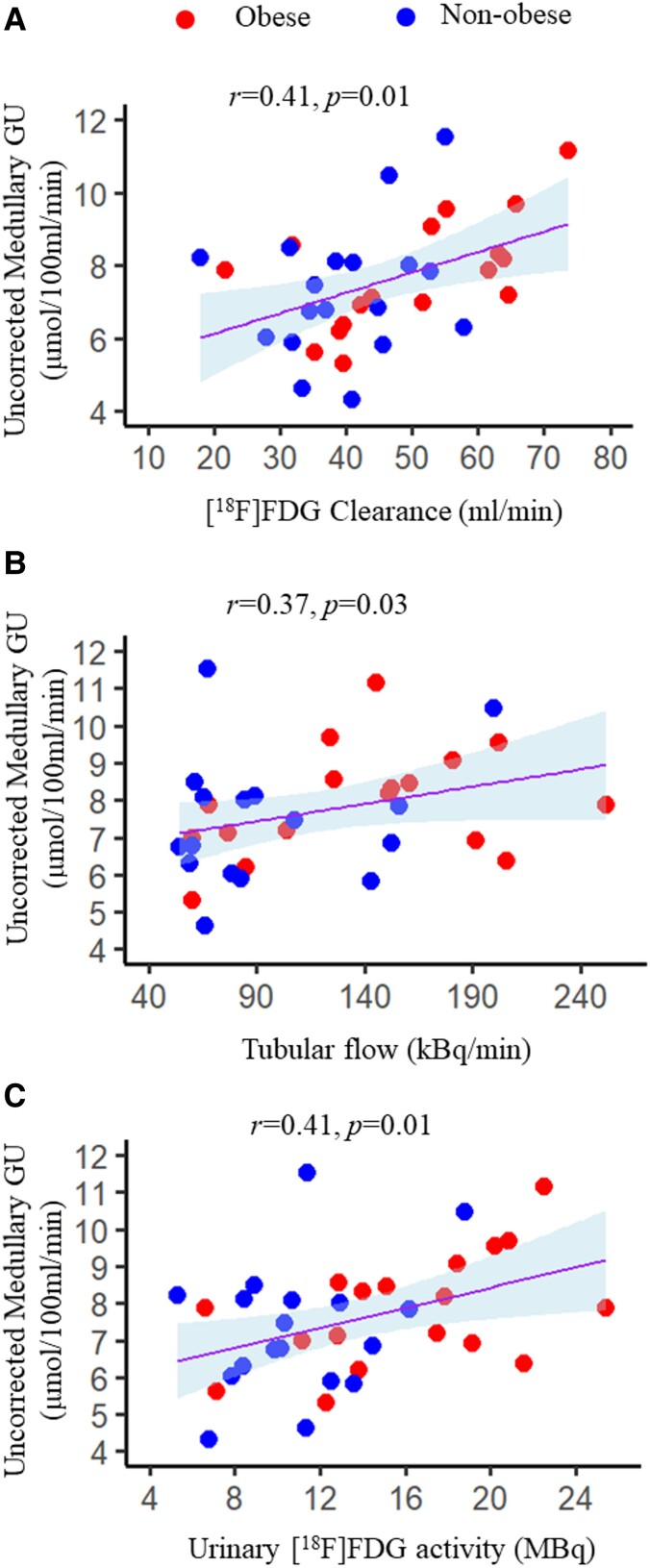
In the pooled data, medullary GU was correlated with the urinary measures (A-C).

Continuous variables are summarized as mean ± SD or median [interquartile range]. The normality of distribution was assessed using the Shapiro-Wilk test. Between-groups comparisons were performed by *t*-test or Mann-Whitney *U* test, as appropriate. Correlations between cortical and medullary GU and clinical parameters and circulating metabolites were performed using either the Pearson correlation coefficient, or Spearman *rho*, as appropriate. For the associations between cortical GU and circulating metabolites, linear multivariate analyses were also performed correcting for the M value. *P* < .05 was considered significant. Analyses were done using JMP version 13.0 (SAS Institute, Cary, NC, USA). Figures 2 and 3 were created using the ggplot package on *R* Studio (18).

## Results

The clinical and metabolic characteristics of the study participants are listed in Table 1. People with obesity and nonobese controls were well-matched for sex and age. As expected, there was a marked difference in adiposity and insulin sensitivity (as indicated by the M value) between the 2 groups. Estimated glomerular filtration rate (mL/min/1.73 m^2^) was similar between the 2 groups.

### Regional Renal Glucose Uptake

Urinary data and regional renal glucose uptake are reported in Tables 2 and 3. Cortical FUR and GU rates tended to be higher in the nonobese controls than in the people with obesity. This difference became significant when correcting the cortical GU data for the intratubular residual [^18^F]FDG. Corrected cortical GU correlated directly with the degree of insulin sensitivity (*r* = .42, *P* = .01) (Fig. 2A), and inversely with BMI (*r* = −0.50, *P* = .002). On the contrary, there was no difference between the 2 groups in either uncorrected or corrected medullary GU rates.

**Table 2.** [^18^F]FDG radioactivity parameters in nonobese and obese subjects

	Nonobese	Obese	*P*
Urine radioactivity (MBq), % of dose	11 [8-13], 6%	16 [12-20], 9%	.003
[^18^F]FDG dose (MBq)	183 [180-189]	183 [175-185]	.8
Urine volume (mL)	606 [536-716]	582 [365-770]	.9
Urinary [^18^F]FDG clearance (mL/min)	40 [33-47]	52 [39-64]	.02
Late plasma activity (kBq/mL)	0.68 [0.52-0.84]	1.15 [0.70-1.90]	.004
Mean urinary flow (mL/min)	4.6 [3.3-6.8]	4.2 [3.0-6.1]	.6
Tubular [^18^F]FDG flow (kBq/min)	73 [59-116]	135 [75-184]	.03
Mean tubular [^18^F]FDG activity (kBq/mL)	27 [19-39]	35 [25-52]	.07
Late tubular [^18^F]FDG activity (kBq/mL)	5.4 [3.8-9.1]	16.2 [8.2-25.1]	.001
Renal tissue [^18^F]FDG clearance (mL/min)	3.2 [2.7-3.7]	3.0 [2.8-3.4]	.5

Entries are median [interquartile range]. *P* value for the difference between obese and nonobese individuals.

Abbreviation: [^18^F]FDG, fluorodeoxyglucose F18.

**Table 3.** Regional renal glucose uptake

	Nonobese	Obese	*P*
Cortical FUR (1/min)	0.01 [0.009-0.012]	0.009 [0.008-0.010]	.08
Cortical GU (µmol/min^−1^/100 mL^−1^)	5.5 [4.3-6.2]	4.6 [4.2-5.2]	.06
Corr. cortical GU (µmol/min^−1^/100 mL^−1^)	4.7 [3.4-5.6]	3.1 [2.2-4.3]	.004
Medullary FUR (1/min)	0.01 [0.012-0.016]	0.016 [0.013-0.017]	.2
Medullary GU (µmol/min^−1^/100 mL^−1^)	7.2 [6.0-8.1]	7.9 [6.8-8.7]	.3
Corr. medullary GU (µmol/min^−1^/100 mL^−1^)	6.4 [5.0-7.4]	6.2 [4.7-7.5]	.8

Entries are median [interquartile range]. *P* value for the difference between obese and nonobese individuals.

Abbreviations: Corr., corrected; FUR, fractional uptake rate; GU, glucose uptake.

### Correlations With FFA and Serum Metabolites

FFA were measured every 60 minutes into the insulin clamp, and the average of these measurements was used. This method has been previously extensively used when assessing the effects of FFA because it decreases their between-measurements variability (19). On the contrary, the other circulating metabolites were measured from a single serum sample taken ∼120 minutes into the clamp. Cortical GU correlated inversely with steady-state FFA (*r* = −0.68, *P* < .0001) (Fig. 2B), and this association remained significant when accounting for the degree of insulin sensitivity (*P* = .003). Cortical GU correlated with a small number of selected metabolites from the NMR metabolomics panel: leucine, isoleucine, valine (branched-chained amino acids), phenylalanine (aromatic amino acid), β-OH-butyrate, acetoacetate (ketone bodies), lactate, and citrate (Table 4). The negative correlations between cortical GU and β-OH-butyrate (*P* = .003), acetoacetate (*P* = .03), and citrate (*P* = .04) remained significant after adjusting for insulin sensitivity.

**Table 4.** Correlations between serum metabolites and cortical glucose uptake

	*rho*	*P*
Leucine	−0.36	.05
Isoleucine	−0.39	.03
Valine	−0.48	.008
Phenylalanine	−0.36	.048
ß-OH-butyrate	**−0**.**58**	.**009**
Acetoacetate	**−0**.**48**	.**008**
Lactate	0.14	.5
Citrate	**−0**.**44**	.**01**
Pyruvate	0.31	.09

Bold entries indicate correlations that remain significant after adjustment for the M value.

## Discussion

There were 2 main findings of the present study. First, we showed that under insulin clamp conditions, renal cortex GU rates were lower in patients with obesity compared with nonobese controls, confirming our previous study (8). Second, we found that cortical GU was negatively associated with circulating steady-state FFA levels (Fig. 2B) and with several circulating metabolites measured under the same experimental conditions (ie, during the insulin clamp) (Table 4). Of these, the negative associations between cortical GU and steady-state FFA, plasma β-OH-butyrate, acetoacetate, and citrate remained significant even after accounting for the degree of insulin sensitivity.

The renal cells of the cortex, and in particular those of the proximal convoluted tubules (PCT), consume large amounts of energy to actively reabsorb solutes—mainly sodium and glucose—from the lumen (20, 21). To meet this high metabolic demand, PCT cells are rich in mitochondria and depend predominantly on oxidative metabolism. Glucose, fatty acids, ketone bodies, lactate, and citrate can all be used as substrates by the PCT (22). Other cells of the nephron are also expected to use glucose. GLUT4 is expressed on podocytes, which are considered insulin sensitive and develop insulin resistance in animal models of diabetes (23, 24), whereas GLUT1 is present in the distal tubuli (25). Thus, the decreased cortical glucose uptake measured with [^18^F]FDG-PET in patients with obesity should reflect a decreased glucose uptake by these cells.

Citrate, the most abundant intermediate of the tricarboxylic acid cycle, is used almost exclusively as a fuel by the kidneys (26). Previous studies using rat kidney cortex slices have shown that citrate inhibits phosphofructokinase (ie, the key enzyme of glycolysis) (27), and that ketone bodies and fatty acids increase gluconeogenesis and decrease glucose uptake through citrate inhibition of phosphofructokinase (28). Moreover, in a study on healthy subjects who underwent renal vein catheterization and infusion of glucose [6-^3^H]glucose and [9,10-^3^H]palmitate under postabsorptive conditions, it was shown that there was an inverse association between renal glucose and FFA uptake, confirming the presence of glucose-FFA competition at the kidney (29).

Our data are in line with these reports and suggest a metabolic pattern such that when circulating citrate, FFA, and ketones are relatively increased—or less suppressed by insulin—renal cortical tissues use less glucose via glycolysis and make more de novo glucose via gluconeogenesis. Our study provides support for the operation of these physiologic pathways in humans in vivo.

It should be mentioned however that with the current methodology it is not possible to assess renal glucose production. When interpreting the [^18^F]FDG-PET results, we should consider 2 important aspects. First, [^18^F]FDG has minimal affinity for SGLT2 (30) and thus [^18^F]FDG would enter the PCT cells via basolateral GLUT2 despite what seems to be the predominant reverse glucose flux. Entry of [^18^F]FDG inside the podocytes or the distal convoluted tubular cells would occur through the same glucose transporters as native glucose. Second, even though the renal cortex comprises different cell types, which the poor spatial resolution of the PET cannot distinguish (31), the PCT cells are the most abundant cells in the renal cortex and they are metabolically very active. They rely predominantly on oxidative metabolism of FFAs as their substrate and possess the enzymatic machinery for gluconeogenesis (9, 32), whereas they have minimal activity of hexokinase (ie, the responsible enzyme for phosphorylation of glucose and [^18^F]FDG) (33). Taken together, a unifying hypothesis of the present findings would be that the lower GU found in patients with obesity is largely to be attributed to an enhanced (or less suppressed) endogenous glucose production because of a higher rate of dephosphorylation by gluconeogenesis, which is known to be generally enhanced in models of insulin resistance (34). Also, [^18^F]FDG uptake, similar to native glucose uptake, could be reduced in podocytes and the distal convoluted tubule via GLUT4 and GLUT1, respectively (25), in patients with obesity. Considering the former conclusion, even though the port of entry of [^18^F]FDG into the PCT cells would differ from that of native glucose, “basolateral” glucose uptake in the proximal tubular cells may have physiological relevance (for instance, under conditions of hyperglycemia), as studies in isolated proximal tubular cells cultured and polarized on porous tissue culture inserts have suggested (35).

We have previously shown that cortical FFA uptake was higher in women with severe obesity compared with healthy lean controls (4). That and the present study show that, in the context of obesity, there is decreased glucose and increased FFA uptake. FFA uptake in the cortex is known to be mediated by the transmembrane glycoprotein CD36 (36, 37), which is highly expressed in renal proximal and distal tubular epithelial cells, podocytes, mesangial cells, microvascular endothelial cells, and interstitial macrophages (37). Importantly, high renal levels of CD36 have been found in patients with chronic kidney disease (CKD) and CD36 has been proposed to play a central role in CKD development (38). In this respect, future larger and prospective studies should evaluate whether an altered renal cortex substrate uptake predicts progression to CKD.

In the present study, we have used the method that we have recently described for correcting renal glucose uptakes rates for the amount of residual [^18^F]FDG within the urinary spaces of the renal tubules (8). The only difference from our previous study was that the acquisition of renal radioactivity was done earlier (ie, at ∼60 minutes from [^18^F]FDG injection). We found that even at this time point, the correction for the residual [^18^F]FDG activity in the urinary space is efficient in “unmasking” the difference in cortical GU between people with obesity and nonobese controls.

On the contrary, whether applying the correction or not, we could not detect any difference in medullary GU rates between the 2 groups. This finding is at variance with our previous report in which we have shown that under insulin clamp conditions medullary GU is higher in lean controls compared with people with obesity (8). The likely explanation for this discrepancy lies with the study protocol. In the present study, the medullary data were associated with urinary clearance of [^18^F]FDG, tubular [^18^F]FDG flow, and urinary [^18^F]FDG activity (Fig. 3), whereas no such association was found with the cortical data. Thus, renal data acquired earlier—at ∼60 minutes from [^18^F]FDG injection—can be reliably used for the assessment of cortical GU, whereas medullary GU seems to be still affected by residual intratubular [^18^F]FDG.

Strength of the present study is the thorough metabolic phenotyping of the study participants using state-of-the-art methods such as PET and NMR metabolomics. Our study also has limitations. First, in the present study, the volume of saline infused was not standardized, though it did not exceed 1.0 L during the whole study. Because aggressive hydration has been previously used to increase the amount of [^18^F]FDG excreted in the urine (39), had this maneuver been applied, our study might have yielded more solid estimates of medullary GU. Moreover, the determination of the hydration status of the study volunteers, the fluid balance, and salt intake (40) may affect regional renal metabolism and a standardized protocol of salt and fluid intake should be applied in future renal PET studies. ROIs were drawn based on anatomical references: a thin band of voxels into consecutive planes were drawn for the cortex just underneath the renal capsule and a parallel inner band for the medulla where the high signal from the pyramids was evident was representative of the medulla. Because the 2 regions are very close to one another and also because of the difficulty of assessing small regions with PET, some interference between the cortical and medullary results cannot be excluded (31). Finally, the decreased cortical GU in patients with obesity does not identify their exact cortical metabolic alterations; preclinical studies will be needed to assess what 2-deoxyglucose “trapping” in each nephron region may suggest for the fate of glucose.

In conclusion, this study confirms that renal cortical GU is higher in healthy nonobese controls compared with people with obesity and shows that cortical GU correlates inversely with circulating substrates such as FFA, ketone bodies, and citrate. These findings are compatible with the concept of substrate competition occurring in the human renal cortex as in skeletal muscle.

## Data Availability

Some or all datasets generated during and/or analyzed during the current study are not publicly available but are available from the corresponding author on reasonable request.

## Clinical Trial Information

The study was registered with www.clinicaltrials.gov (ID no. NCT04343469).

## Abbreviations

18F FDG: BMI, body mass index
CKD: chronic kidney disease
FFA: free fatty acid
FUR: fractional uptake rate
GU: glucose uptake
NMR: nuclear magnetic resonance
PCT: proximal convoluted tubule
PET: positron emission tomography
ROI: region of interest
T2D: type 2 diabetes
